# Consequences of a Missed History: A Case of Antidepressant Discontinuation Syndrome

**DOI:** 10.7759/cureus.10950

**Published:** 2020-10-14

**Authors:** Sajid Hameed, Mukesh Kumar, Piyush Puri, FNU Sapna, Pal Satyajit Singh Athwal

**Affiliations:** 1 Neurology, Aga Khan University, Karachi, PAK; 2 Neurology, Shaheed Mohtarma Benazir Bhutto Medical University, Larkana, PAK; 3 Internal Medicine, Al-Falah School of Medical Sciences and Research Center, Faridabad, IND; 4 Internal Medicine, Lal Medical Center, Larkana, PAK; 5 Internal Medicine, Saraswathi Institute of Medical Sciences, Hapur, IND

**Keywords:** anti depressants, withdrawls, headache, diarrhoea, depression, taper

## Abstract

Antidepressant discontinuation syndrome (ADDS) is reported to occur in almost 30-50% of the patients who take antidepressants for a duration of at least four to six weeks and then suddenly discontinue the drug. Since there is an increase in the use of antidepressants for various reasons by general practitioners, patient education about when and how to discontinue a drug is not acknowledged enough. It is reported to occur with the use of different classes of antidepressants - selective serotonin reuptake inhibitor (SSRI), monoamineoxidase inhibitor (MAOI), tricyclic antidepressants (TCAs), and atypical antipsychotics like risperidone, trazodone, clozapine, and venlafaxine. Slow tapering off the drugs has also caused ADDS. Symptoms start within two to four days of quitting the drug and are usually mild lasting for two to four weeks (can persist for six to 12 months) but could be severe enough leaving the patient nonambulatory. Here, we represent a case of a 55-year-old female who presented to the outpatient clinic with complaints of headache, vomiting, and diarrhea. The patient had 10 to 12 episodes of watery diarrhea every day and bilateral, continuous, pressing headache associated with multiple episodes of non-projectile vomiting. She was investigated for ultrasound sonography (USG) abdomen, CT head, and lab investigations which turned around to be normal. A follow-up visit with detailed history revealed she suddenly stopped taking escitalopram after six months by herself without tapering off the dose, two days before the onset of symptoms. Escitalopram was reinstated and the symptoms started to resolve in two to three days. All the unnecessary investigations and treatment could have been prevented if the proper history was taken and revealed at the initial visit.

## Introduction

Depression is of prime importance to the overall global burden of diseases. Globally, it affects more than 264 million people of all age groups [1]. Antidepression drugs are used not only for depression but sundry other disorders like general anxiety disorder (GAD), obsessive-compulsive disorder (OCD), eating disorders, post-traumatic stress disorder (PTSD), schizophrenia, neuropathic pain, etc. and hence there is an upsurge in the use of typical and atypical antidepression drugs from a decade ago [2]. New cases of depression are twice as high in women than men in the younger age group (14-25 years) [3]. 

Antidepressant discontinuation syndrome (ADDS) was first reported with the use of imipramine (a tricyclic antidepressant) shortly after it was first clinically used [4]. Symptoms of discontinuation syndrome are reported to occur more in drugs with a shorter half-life than in drugs with a longer half-life [5]. A mnemonic, FINISH, has been devised to recognize clinical symptoms of ADDS so that it is not left obscured: F - Flu-like symptoms (headache, dizziness), I - Insomnia, N - Nausea, I - Imbalance, S - Sensory Disturbance, H - Hyperarousal, with other clinical features like electric shock-like sensations (brain zap), myalgia, insomnia, blurred vision, amnesia, disorientation, diarrhea, depersonalization, depressive mood, increased suicidal thoughts, etc., which could mimic rebound depression [6].

Mild symptoms usually lead to misdiagnosis and unnecessary investigations and treatment. Education about this common and likely underrecognized clinical phenomenon will help prevent future episodes and minimize the risk of misdiagnosis. Patients are enticed to discontinue their drugs once they start feeling better and this could lead to an early relapse of the illness as well as increased chances of ADDS. Here, in this case, we emphasize the importance of detailed history taking and early diagnosis of ADDS, which should be managed at the earliest else it leads to misdiagnosis with unnecessary interventions.

## Case presentation

We present a case of a 55-year old female who presented to the outpatient clinic with a three-day history of abdominal cramps, headache, and diarrhea. The headache was continuous, bilateral diffuse, pressing in character, moderate in intensity with intermittent severe exacerbations. It was associated with nausea and multiple episodes of non-projectile vomiting. There was no history of fever, photophobia, phonophobia, lacrimation, vision changes, weight loss, joint pain, limb weakness, or numbness. There was no history of similar headaches in the past. The patient had watery diarrhea about 10 to 12 times a day. There was no pus or blood in it. On past medical history, she had been diagnosed with a generalized anxiety disorder for which she was prescribed escitalopram (an SSRI), which she had stopped after taking for six months. There was no history of smoking, alcohol, or illicit drug use. She was married with four children. 

**Figure 1:**
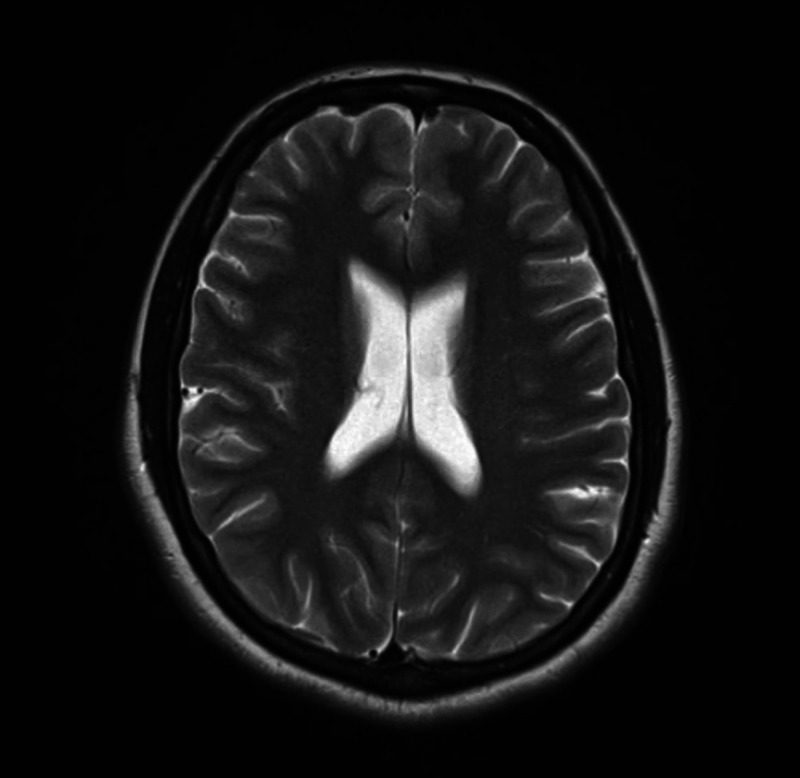
Patient's normal MRI cranium No ventriculomegaly was seen. No focal, hyperdense/hypodense lesions were seen.

**Figure 2:**
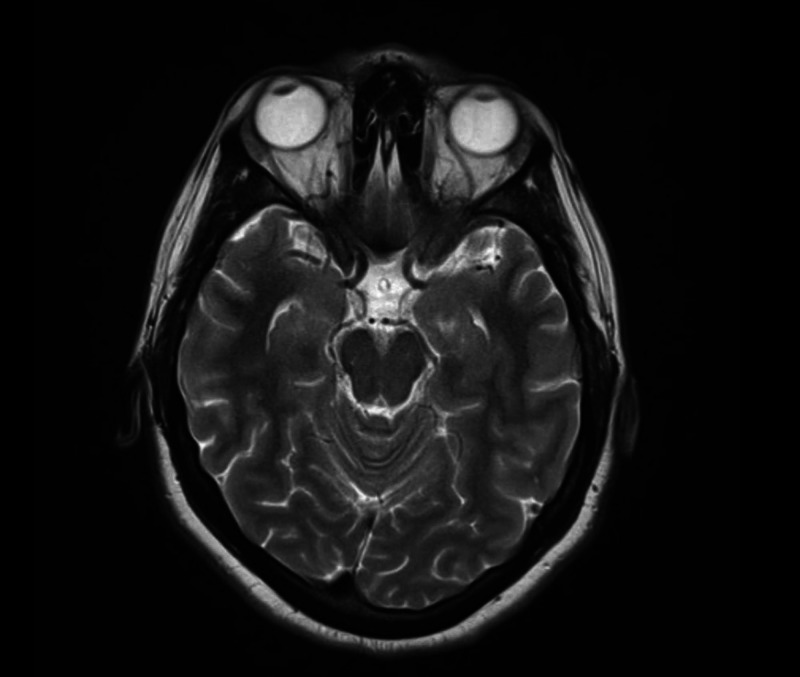
Normal MRI of the skull

The physical examination was within normal limits, except exaggerated bowel sounds. Meningeal signs were absent. There was no fever or papilledema. She was investigated and everything came out normal, including cranial magnetic resonance imaging (MRI) (Figure 1, 2), ultrasound abdomen, and baseline laboratory investigations including the liver function tests and blood cultures.

On a follow-up visit after four days, a repeat drug history revealed that she has suddenly stopped escitalopram by herself without tapering the dose, only two days preceding these symptoms. We restarted escitalopram and all her symptoms were resolved within three days. A diagnosis of antidepressant discontinuation syndrome was made.

## Discussion

The pathophysiology of ADDS is still largely unknown and the symptoms affecting sleep, balance, gastrointestinal tract, and sensory system are thought to be due to an imbalance among the norepinephrine, cholinergic, and serotonin neurotransmitters [7]. It is by and large related to the half-life of the drug and the patient’s rate of metabolism, with symptoms more reported on discontinuation of paroxetine than fluoxetine (paroxetine has a shorter half-life than fluoxetine) [8]. Some researchers have suggested the role of genetics in the pathophysiology [9]. A finding in a clinical study shows the involvement of C(-1019) G polymorphism of the serotonin 5-HT1A receptor gene in the occurrence of paroxetine discontinuation syndrome [9]. The newer antidepressant medications that are used for anxiety disorder without any supporting evidence in the efficacy of antidepressants have a similar dependence problem to benzodiazepines, although benzodiazepines are used much less for treating anxiety disorders, studies have shown that benzodiazepines have better efficacy in controlling panic attacks and cause less antidepression discontinuation syndrome symptoms and hence the role of the benzodiazepines should be thoroughly reexamined [10].

Some researchers have reported that ADDS occurs more in patients with early-onset dysthymic syndrome and it tends to affect females more [11]. Also, reports suggest that there is no difference between discontinuation symptoms when antidepressants are used for different disorders like depression and anxiety disorder and reports also suggest that longer duration of treatment is not directly related to more severe discontinuation syndrome symptoms [12].

Increased off-label use of antidepressants like in sexual dysfunction, insomnia, migraine, and premenstrual syndrome, especially by primary care physicians, is associated with an increased incidence of antidepression discontinuation symptoms [13]. Multiple case reports demonstrate a similarity between the symptoms of ADDS caused by SSRI and TCA but the problems related to movement (Parkinsonism), sleep disturbances, and GI symptoms are more closely related to the discontinuation of TCAs [14].

## Conclusions

This case report highlights the importance of differential diagnosis of antidepressant discontinuation syndrome (ADDS) even when no psychiatric symptoms are present in the patient, since the use and prescription of antidepressant drugs has soared high in the last decade. It also highlights the importance of a detailed history taking. As in our case, a detailed drug history on the first visit could have avoided unnecessary investigations and follow-up. Also, when prescribed an antidepressant, the patient should be well educated about the disease, drug dosage, and relapse/withdrawals on abrupt discontinuation.
